# Synergistic Effects of Nonthermal Plasma and Disinfecting Agents against Dental Biofilms *In Vitro*

**DOI:** 10.1155/2013/573262

**Published:** 2013-09-12

**Authors:** Ina Koban, Marie Henrike Geisel, Birte Holtfreter, Lukasz Jablonowski, Nils-Olaf Hübner, Rutger Matthes, Kai Masur, Klaus-Dieter Weltmann, Axel Kramer, Thomas Kocher

**Affiliations:** ^1^Unit of Periodontology, Department of Restorative Dentistry, Periodontology and Endodontology, Ernst-Moritz-Arndt University Greifswald, Walther-Rathenau-Straße 49a, 17487 Greifswald, Germany; ^2^Institute for Medical Informatics, Biometry and Epidemiology, University of Duisburg-Essen, Hufelandstrasse 55, 45122 Essen, Germany; ^3^Institute for Hygiene and Environmental Medicine, Ernst-Moritz-Arndt University Greifswald, Walther-Rathenau-Straße 49a, 17475 Greifswald, Germany; ^4^Robert Koch Institut, Nordufer 20, 13353 Berlin, Germany; ^5^Leibniz Institute for Plasma Science and Technology, Felix-Hausdorff-Straße 2, 17489 Greifswald, Germany

## Abstract

*Aim*. Dental biofilms play a major role in the pathogenesis of many dental diseases. In this study, we evaluated the synergistic effect of atmospheric pressure plasma and different agents in dentistry on the reduction of biofilms. *Methods and Results*. We used monospecies (*S. mutans*) and multispecies dental biofilm models grown on titanium discs *in vitro*. After treatment with one of the agents, the biofilms were treated with plasma. Efficacy of treatment was determined by the number of colony forming units (CFU) and by live-dead staining. For *S. mutans* biofilms no colonies could be detected after treatment with NaOCl or H_2_O_2_. For multispecies biofilms the combination with plasma achieved a higher CFU reduction than each agent alone. We found an additive antimicrobial effect between argon plasma and agents irrespective of the treatment order with cultivation technique. For EDTA and octenidine, antimicrobial efficacy assessed by live-dead staining differed significantly between the two treatment orders (*P* < 0.05). *Conclusions*. The effective treatment of dental biofilms on titanium discs with atmospheric pressure plasma could be increased by adding agents *in vitro*.

## 1. Introduction

Plasma is the fourth state of matter besides the solid, liquid, and gaseous states. It is an ionized gas consisting of ions, a broad spectrum of radicals, ultraviolet irradiation, electric fields, and ozone, which are responsible for its antimicrobial efficacy [1].

Atmospheric pressure plasma is also called nonthermal plasma, because it can achieve body temperature [2]. This allows medical application to humans by small plasma hand device on humans [3]. Plasma medicine is a new scientific field, and many research groups investigated various applications, for example, to the treatment of dental diseases like periodontitis [4], peri-implantitis [5], and caries and denture stomatitis [6] as well as dermatological diseases and chronic wounds [1, 7, 8]. Most applications were based on the antimicrobial effect of plasma to disinfect the skin, implants, and other medical devices. To date antiseptics have been commonly applied in these cases. 

Plasma is especially interesting for fields with a dissatisfactory standard therapy or where an effective therapy does not exist, for example, peri-implantitis therapy in dentistry. The antimicrobial efficacy of plasma could be increased by raising the electrical input power [9, 10]. However, plasma should also be tissue tolerable and applicable to humans. As with any active substance the balance between efficacy and tolerability needs to be found.

To enhance plasma effects without an increased input power, we investigated possible synergistic effects between atmospheric pressure plasma and different antiseptics including chlorhexidine, octenidine, and polyhexanide as well as the chemicals sodium hypochlorite and hydrogen peroxide, often used in dental practice, and the chelating agent EDTA.

## 2. Material and Methods

We used three different dental biofilm models grown on titanium, the typical dental implant material. Titanium discs were machined and had a diameter of 5 mm and 1 mm thickness (Straumann, Basel, Switzerland). 

### 2.1. Monospecies Biofilm *Streptococcus mutans*

*Streptococcus mutans* (*S. mutans* DSM 20523, German collection of microorganisms and tissue culture cells, Braunschweig, Germany), a strain often utilized for antimicrobial tests, was grown overnight at 37°C on Columbia sheep blood agar (BBL, BD, Heidelberg, Germany). One inoculation loop of this culture was resuspended into 30 mL Brain Heart Infusion (BHI) (BD, BBL, Heidelberg, Germany) complemented with 1% sucrose [11]. The sterile titanium discs were positioned in 96-well microtiter plates (Techno Plastic Products AG, Trasadingen, Switzerland), covered with 100 *μ*L *S. mutans* suspension, and incubated aerobically at 37°C. For *S. mutans*, we deliberately used no surface coating with salivary proteins, since this reduces the contact angle of titanium, and, consequently, adhesion of *S. mutans* would be reduced [12]. In preliminary tests we achieved the best biofilm forming results without a conditioning film. Every 24 h BHI was changed. After 72 h the medium was drawn off, the discs were washed with 0.9% NaCl solution and transferred into a new, sterile microtiter plate.

### 2.2. Multispecies Saliva Biofilm

To simulate a perimucositis situation, we used a multispecies saliva biofilm. Unstimulated saliva was taken from healthy donors (*n* = 5, pooled saliva, mean age 29 ± 7 years, nonsmokers) as a source of oral microbiota. This was approved by the local ethics committee (BB 120/10). Saliva donors did not take any medication three months prior to the study and did not have active carious lesions or overt periodontal disease. In the saliva of three donors we found *Aggregatibacter actinomycetemcomitans* and *Porphyromonas gingivalis*, and *Fusobacterium nucleatum* in four donors and *Streptococcus sanguinis* in all five donors in a PCR analysis. These bacteria are typically associated with periodontitis, perimucositis, or peri-implantitis.

Sterile titanium discs were covered with 100 *μ*L saliva and incubated aerobically at 37°C. We deliberately used no previous surface coating with salivary proteins, because we inoculated full saliva containing all proteins. The further procedure was as described above.

### 2.3. Multispecies Subgingival Biofilm

To study possible synergistic effects in an anaerobic milieu, we used a multispecies subgingival biofilm. Subgingival plaque was obtained from five periodontal patients (pooled plaque, mean age 50 ± 10 years). It was removed with a dental curet and resuspended in Schädler broth with vitamin K1 (BD, BBL, Heidelberg, Germany). The plaque was incubated at 37°C overnight under anaerobic conditions using the Anaerocult A system (Merck, Darmstadt, Germany) to develop a biofilm. Before biofilm formation the sterile titanium discs were conditioned with DTT saliva. To this end, 10 mL saliva was collected from healthy donors, pooled, and treated with 5 mL 1 M DL-dithiothreitol solution (DTT, Sigma-Aldrich, Steinheim, Germany) and 5 mL distilled water. This DTT saliva mixture was then centrifuged (Mini Spin, Eppendorf, Hamburg Germany). Thereafter the supernatant was filtered through a 0.2 *μ*m pore filter (HVM Filtramed, Rotenburg an der Fulda, Germany) and frozen at −20°C until use. The titanium discs were covered with 500 *μ*L DTT saliva and incubated 2 h at 37°C. Afterwards the DTT saliva was carefully removed, and 1 mL plaque suspension was added and incubated anaerobically at 37°C.

### 2.4. Antiseptic and Chemical Pretreatment

Titanium discs were pretreated with 0.1% chlorhexidine digluconate (CHX, aqueous solution, Fagron GmbH & Co. KG, Barsbüttel, Germany), 0.1% octenidine dihydrochloride (OCT, Schülke & Mayr GmbH, Norderstedt, Germany), 0.1% polihexanide (PHMB, B. Braun, Melsungen, Germany), 0.6% sodium hypochlorite (NaOCl, AppliChem GmbH, Darmstadt, Germany), 1.5% hydrogen peroxide (H_2_O_2_, AppliChem GmbH, Darmstadt, Germany), and 20% ethylenediaminetetraacetic acid (EDTA, AppliChem GmbH, Darmstadt, Germany). As negative control we used 0.9% NaCl solution, hereafter called “NaCl control.” 

Biofilm discs were covered with 100 *μ*L of the substances and incubated for 30 min. Within this incubation time, antiseptically pretreated biofilms were treated 1 min with plasma. Afterwards the antiseptic effect was halted by adding 1 mL inactivator. For inactivating PHMB effects we used 30 g/L Tween 80, 30 g/L saponin, 1.0 g/L histidin, and 1.0 g/L cystein. For CHX, OCT, and EDTA, the inactivator was Lipofundin MCT 20% (B. Braun, Melsungen, Germany). An inactivator consisting of 30 g/L Tween 80, 3 g/L lecithin, 1 g/L histidin and 5 g/L sodium thiosulfate was used to stop the effect of NaOCl and H_2_O_2_. All inactivators were proven by the quantitative suspension test according to DIN EN 1040 (German Institute for Standardisation).

### 2.5. Plasma Treatment

To test the resistance of antiseptics against plasma treatment, we treated 5 mL of each antiseptic for 1 and 10 min with plasma. By spectral measurement (CHX: 231 nm, OCT: 213 nm, PHMB: 235 nm) we compared measurement results after plasma treatment with untreated antiseptics. 

For plasma generation we used the atmospheric pressure plasma jet kINPen 09 (neoplas tools, Greifswald, Germany) [8]. Argon (Ar) gas flow was set to 5 slm (standard liters per minute). The flow rate was controlled by a flow controller (MKS Instruments, Munich, Germany). We had a constant pin-to-disc distance of 7 mm during the application. The kINPen was fixed in a computer driven 3 axes (*x*, *y*, *z*) motorized stage, under which a 96-well plate with the titanium discs was positioned. The plasma devices were consecutively driven from well to well, positioned centrally over the discs and remained in position for 1 min. To assess the effects of biofilm dehydration by gas flow, biofilms were treated with Ar gas without plasma generation. Additionally we investigated biofilms without gas or plasma treatment, hereafter called “control procedure.”

After plasma treatment, titanium discs were placed into wells with 200 *μ*L 0.9% NaCl solution and the biofilm was removed by ultrasonic scaling. Serial dilutions of the resuspended biofilm solution were made by transferring 0.1 mL of the resultant suspension to 0.9 mL of fresh 0.9% NaCl solution. Afterwards an aliquot portion of 0.1 mL from each dilution was plated on Columbia sheep blood agar (BBL, BD, Heidelberg, Germany) and incubated aerobically at 37°C for 48 h for *S. mutans* and saliva. Resuspended plaque biofilms were plated on Schädler agar (BBL, BD, Heidelberg, Germany) and incubated anaerobically at 37°C for 48 h. The colonies were counted and expressed as colony forming units (CFU/mL). 

### 2.6. Change in Treatment Order

In an additional experiment we changed the treatment order because we wanted to test the hypothesis that EDTA destroys the biofilm matrix to increase the plasma effect and that a higher antimicrobial effect after an increased radical induction of H_2_O_2_ by plasma treatment is observable. First saliva biofilms were treated with plasma, and then EDTA or H_2_O_2_ was added. After 30 min the antiseptic effect was halted by adding 1 mL inactivator as described above. Then, CFUs were determined. 

### 2.7. Live-Dead Staining

Biofilms on discs were stained with fluorescein diacetate (FDA) and ethidium bromide (EB) to selectively stain living (green) and dead (red) bacteria [13]. Thereby 6 *μ*L of a FDA stock solution (5 mg/mL) and 3 *μ*L of an EB stock solution (1.25 mg/mL) were mixed in 1 mL 0.9% NaCl solution. The fluorescence was measured at excitation wavelengths of 485 nm and 530 nm and emission wavelengths of 530 nm and 630 nm on Berthold TriStar LB 941 (Berthold Technologies, Bad Wildbad, Germany). The division of the green fluorescent signal by the red fluorescent signal yielded the live-dead ratio. With the live-dead staining we tested the antimicrobial effect with all agents in both treatment orders on saliva biofilms. Additionally, samples were observed using a Zeiss CLSM510 Exciter confocal laser scanning microscope (Carl Zeiss MicroImaging GmbH, Jena, Germany). Using Live-dead staining the antimicrobial effect was tested with all agents in both treatment orders on saliva biofilms. 

### 2.8. Statistics

For all analyses, observed CFU values were transferred to their base 10 logarithm (referred to as log_10_CFU; for *S. mutans*: log⁡_10_(CFU + 1)). Continuous data are presented as mean ± standard deviation (SD).

The log⁡_10_-reduction factor (RF) for each treatment method was calculated [14].

To examine differences in log_10_CFU values between different procedures and admixture combinations, log_10_CFU values were compared for each agent versus the NaCl control within each procedure (within columns of Table 1) and for each agent; differences between the NaCl control, Ar gas, and Ar plasma were pairwise evaluated (within rows of Table 1) using two-sided Mann-Whitney *U* tests. To adjust for multiple testing within each step (comparisons within columns, resp., within rows), *P* values were corrected according to Bonferroni.

Differences in log_10_CFU values across different procedures (control procedure, Ar gas, and Ar plasma) and agents (NaCl control, CHX, OCT, PHMB, NaOCl, H_2_O_2_, and EDTA) were evaluated using linear regression analyses including the twofold interaction term of both factors (significant at *P* < 0.10). Linear regression coefficients with their 95% confidence intervals (CI) were reported. *R* ^2^ quantifies the amount of variation explained by the model.

Differences of live-dead ratios across combinations of procedures and admixtures were evaluated using two-sided Mann-Whitney-*U*-tests. *P* values were corrected according to Bonferroni.

Statistical differences were considered significant at a level alpha of 5%. Statistical analyses were performed with STATA/SE 10.0 (Stata Corp LP, College Station, TX, USA) and R 2.13.0 (R Development Core Team, 2011).

## 3. Results

The measured spectral data of the antiseptics before and after plasma treatment were identical (OCT: 0.5, CHX: 1, PHMB: 1.1), indicating that the agents were not modified by plasma.

### 3.1. Log_**10**_CFU Values after Administration of Agent Plus Plasma/Gas

#### 3.1.1. *S. mutans* Biofilm

The highest CFU reduction with no colonies being detected (values below detection limit) was achieved for six combinations (Table 1): NaOCl with any procedure and H_2_O_2_ with any procedure.

Compared with the control procedure, all agents lead to significantly lower CFU values compared with the negative control (*P* < 0.05). In combination with Ar gas, admixture of CHX (RF = 1.96), OCT (RF = 1.90), PHMB (RF = 3.39), NaOCl (RF = 6.91), and H_2_O_2_  (RF = 6.91) significantly reduced CFU values compared with NaCl controls. In combination with Ar plasma, admixture of PHMB (RF = 3.36), NaOCl (RF = 6.91), H_2_O_2_  (RF = 6.91), and EDTA (RF = 2.78) significantly reduced CFU values compared with NaCl controls.

Compared with the control procedure, only Ar plasma significantly reduced log_10_CFU values when combined with NaCl control, PHMB, or EDTA (*P* < 0.05). For admixtures of CHX, OCT, NaOCl, and H_2_O_2_, log_10_CFU values did not differ significantly across procedures. For NaCl control and EDTA, log_10_CFU values were significantly reduced for Ar plasma compared with Ar gas (*P* < 0.05).

To evaluate the impact of procedure and agents on *S. mutans* CFU values, linear regression models were evaluated (Table 2). The model explained 98.4% of the variation in observed CFU values. Procedure, admixture, and the interaction of both were significantly related to log_10_(CFU/mL) values (*P* < 0.001). Post hoc analysis confirmed that any combination with NaOCl or H_2_O_2_ was significantly the best performing combination among those tested (*P* < 0.001) with a predicted reduction of 7.51 log_10_(CFU/mL) compared with NaCl control.

#### 3.1.2. Saliva Biofilm

The highest reduction in log_10_CFU was found for the combination of Ar plasma with NaOCl (RF = 3.06, Table 1). Compared with the control procedure, admixture of OCT, PHMB, NaOCl, H_2_O_2_, and EDTA led to significantly lower CFU values compared with the NaCl control (*P* < 0.05). Combined with Ar gas, admixture of CHX, OCT, NaOCl, and H_2_O_2_ significantly reduced CFUs compared with NaCl control (*P* < 0.05). The effect of Ar plasma on CFU reduction was significantly enhanced by OCT, NaOCl, and EDTA compared with the NaCl control.

Compared with the control procedure, application of Ar gas significantly reduced CFU values when CHX or OCT was applied previously (*P* < 0.05). Compared with the control procedure, Ar plasma treatment significantly enhanced antimicrobial effects of NaCl control, CHX, OCT, PHMB, and EDTA (*P* < 0.05). For NaCl control, OCT and EDTA, Ar plasma performed significantly better compared with Ar gas (*P* < 0.05).

For the combination of plasma and agents, the simple additive effect on log_10_CFU reduction was partly exceeded. Compared with single reduction factors for plasma and CHX (RF = 1.48 and RF = 0.28, resp.), the combined RF was 1.70 (Table 1).

Estimating the impact of procedure and agents on saliva log_10_CFU values more closely (Table 2), linear regression models were performed, which explained 72.5% of the variation in observed CFU values. Procedure, admixture, and the interaction of both were significantly related to log_10_CFU values (*P* < 0.001). Post hoc analyses revealed that the combination of Ar plasma with NaOCl was the best performing combination among those tested with a predicted reduction of 3.07 log_10_CFU compared with the total negative control (NaCl control with control procedure). It was significantly better compared with most combinations but similarly effective compared with Ar gas combined with NaOCl (*P* = 0.52), Ar plasma combined with OCT (*P* = 0.22), and Ar plasma combined with EDTA (*P* = 0.38). 

#### 3.1.3. Subgingival Biofilm

The highest log_10_CFU reduction was found for Ar plasma with H_2_O_2_ (4.41 ± 0.86, Table 1). For the control procedure any admixture led to significantly reduced log_10_CFU values compared with NaCl control (*P* < 0.05). With Ar gas, only the admixture of OCT was beneficial (log_10_CFU 4.68 ± 1.53). Regardless of the admixture, combinations with Ar plasma led to comparable reductions in log_10_CFU values.

Considering the effect of the three procedures within agents, both Ar gas and Ar plasma significantly reduced log_10_CFU values compared with the control procedure for NaCl control and H_2_O_2_. For PHMB, NaOCl, and EDTA, log_10_CFU values did not differ significantly across procedures. For NaCl control and H_2_O_2_, log_10_CFU values were significantly reduced for Ar plasma compared with Ar gas. The overall best log_10_CFU reduction was achieved when Ar plasma was combined with H_2_O_2_  (RF = 2.92).

To evaluate the impact of procedure and agents on plaque log_10_CFU values, linear regression models were evaluated (Table 2). While 76.3% of the variation in observed log_10_CFU values were explained, procedure, admixture, and the interaction of both were significantly related to log_10_CFU values(*P* < 0.001). Post hoc analyses revealed that the combination of Ar plasma with H_2_O_2_ was significantly the best performing combination among those tested (*P* < 0.001) with the exception of Ar gas with OCT (*P* = 0.25). The combination of Ar plasma with H_2_O_2_ had a predicted reduction of 2.92 log_10_CFU compared with the total negative control (NaCl control with control procedure).

### 3.2. Log_**10**_CFU Values after Administration of Plasma Plus Agent 

To evaluate whether the reduction in CFU was related to the procedure or the admixture, the treatment order was changed; that is, plasma or gas was applied before the admixture of agents (Table 3). The data were comparable with those of Table 1. The extent of log_10_CFU reduction relative to the total negative control was comparable, irrespective of the treatment order. For example, the combination of EDTA plus Ar plasma achieved a log_10_CFU reduction of RF = 2.81, while the combination of both in reversed treatment order achieved a log_10_CFU reduction of RF = 2.66. For the combination of H_2_O_2_ with Ar plasma, respective log_10_CFU reductions were 2.41 and 2.58. Accordingly, we found an additive effect of the antimicrobial effects of plasma (RF_plasma_ = 1.35) and H_2_O_2_ (RF_H_2_O_2__ = 1.00) when combined: RF_plasma+H_2_O_2__ = 2.58 (>1.35 + 1.00). The same figures applied to the combination of plasma (RF_plasma_ = 1.35) with EDTA (RF_EDTA_ = 1.25) and plasma + EDTA: RF_plasma+EDTA_ = 2.66(~1.35 + 1.25).

### 3.3. Live-Dead Staining

**Figure 1:**
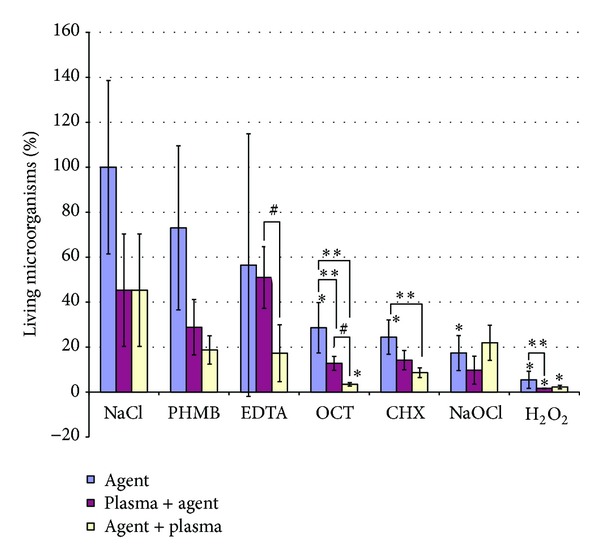
Green fluorescence (fluoresceindiacetate (FDA); alive): red fluorescence (ethidiumbromide (EB); damaged membrane dead) ratio of saliva biofilms after treatment with different procedures. Data are presented as mean ± SD (*n* = 8). *P < 0.05 versus NaCl control (within equally colored columns); **P < 0.05 versus Agent only for each agent separately; ^#^ *P* < 0.05 Plasma plus Agent versus Agent plus plasma for each agent separately. *P* values were retrieved from Mann-Whitney-*U* tests and were Bonferroni corrected.

To determine the antimicrobial effect of all combinations with both treatment orders, we stained the biofilms with EB and FDA (Figure 1). A reduction of the living microorganisms in % can be explained by a reduced FDA fluorescence or an increased EB fluorescence signal, resulting in a higher number of dead cells. The negative control achieved a mean value of 100%. For Ar plasma treated cells the value decreased to 45.3%. After EDTA treatment the mean living microorganisms of 56.4% were further reduced to 17.3% after additional plasma treatment. When EDTA treatment was applied after Ar plasma treatment, no additive effect was found (51%). Both values differed significantly between the two treatment orders (*P* < 0.05). 

Concerning antiseptics, additional Ar plasma treatment consistently decreased the mean ratio with only minimal differences between either treatment order (CHX: 24.4%, CHX + Ar plasma: 8.6%, and Ar plasma + CHX: 14.2%; OCT: 28.6%, OCT + Ar plasma: 3.4%; Ar plasma + OCT: 14.17; PHMB: 73%; PHMB + Ar plasma: 18.7%, Ar plasma + PHMB: 28.8%). Applying the antiseptic treatment before Ar plasma treatment led to slightly fewer living microorganisms compared with the other treatment order. This difference was significant (*P* < 0.05) for OCT. For NaOCl, the lowest ratio was achieved when plasma treatment was applied prior to NaOCl treatment (17.4%; 21.9% when Ar plasma treatment was applied after NaOCl treatment). The highest reduction was achieved by H_2_O_2_ (5.4%). With prior (posterior) Ar plasma treatment the ratio was 1.6% (2.2%).

**Figure 2:**
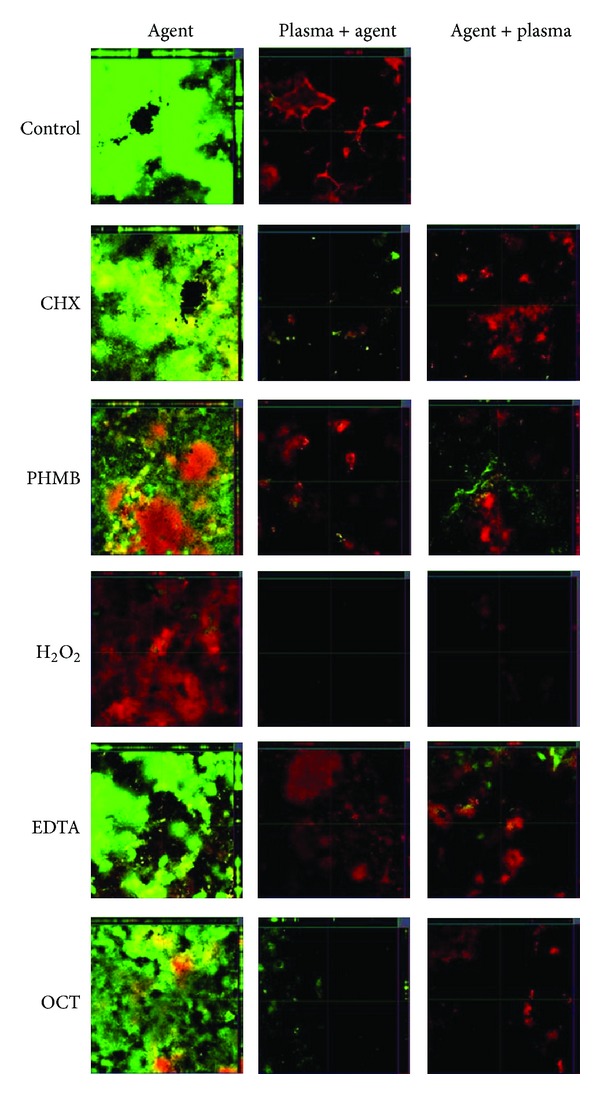
Fluorescence staining ofsaliva biofilms on titanium discs for different treatments: EDTA, EDTA plus Ar Plasma, Ar Plasma plus EDTA, H_2_O_2_, H_2_O_2_ plus Ar plasma and Ar plasma plus H_2_O_2_. The biofilms were stained with fluoresceindiacetate (FDA) and ethidiumbromide (EB) to selectively stain living (green) and dead (red) bacteria.

Using confocal laser scanning microscopy (Figure 2) the difference between the H_2_O_2_ treatments was more pronounced in the center of the disc, where no green fluorescence was visible. The micrographs for both treatment orders for the combination of EDTA and Ar plasma were comparable. Using the same microscopic settings the micrographs of Ar plasma and antiseptic after treated biofilms were comparable to those of EDTA and Ar plasma. Therefore the pictures have not been presented here. 

## 4. Discussion

In this study we found an additive antimicrobial effect between Ar plasma and agents irrespective of the treatment order regarding cultivation. Concerning Live-dead staining we found different antimicrobial effects dependent on treatment order.

Decontamination of dental implants is a promising application of plasma devices. To investigate synergistic effects between Ar plasma and agents, we used three different biofilm models. 

In this study, sensitivity to treatments differed significantly between our biofilm models. Monospecies *S. mutans* biofilms were more susceptible to antimicrobial treatments than our multispecies biofilm models. *S. mutans* biofilm is no satisfactory model to test antibiofilm efficacy, because it cannot mimic oral conditions in an adequate way. Therefore we used biofilms cultured from *ex vivo* saliva and subgingival dental plaque to test Ar plasma in more realistic models. It was shown that Gram-negative as well as anaerobic bacteria are more sensitive to plasma than Gram positives or aerobic ones [15]. In multispecies saliva and subgingival biofilms we found both Gram-negative and -positive bacteria. We detected the highest CFU reduction by Ar plasma for the subgingival plaque biofilm. However, during biofilm processing we could not maintain the anaerobic atmosphere, which may have distorted the antimicrobial effect of the treatments. 

Using only culture-based techniques to determine antimicrobial effects bears some disadvantages. Bacteria can exist in a viable but nonculturable state (VBNC) [16]. VBNC bacteria cannot be detected using culture-based techniques. Additionally, not all species in our multispecies biofilm grow on sheep blood agar. Therefore we used Live-dead staining with a microscopic and a spectroscopic analysis in saliva biofilms. This method showed more differences between treatment regimes. 

In this study, we found additive effects between Ar plasma and agents. To understand the magnitude of these effects, we compared the CFU and live-dead ratio reduction of Ar plasma and the agents and the CFU and live-dead-ratio reduction of the combination of both. 

Concerning culture-based technique, the antibiofilm effect of Ar plasma was significantly increased for *S. mutans* biofilms by adding EDTA. No colonies were detectable after NaOCl or H_2_O_2_ treatment. With anaerobic multispecies biofilms we found significant synergistic effects between H_2_O_2_ and Ar plasma, which was also the procedure with the highest log_10_CFU reduction of saliva biofilm. For aerobic multispecies saliva biofilms significant additive effects between Ar plasma and EDTA and OCT were found. Log_10_CFU reductions for combined treatments were always higher than single Ar plasma or single agent treatments. 

Treatment order differed significantly between addition of EDTA and octenidine before or after plasma application (*P* < 0.05) when we evaluated the results with Live-dead staining. When EDTA treatment was applied after Ar plasma treatment, no additive effect was found. Concerning antiseptics, additional Ar plasma treatment consistently decreased the mean ratio irrespective of the treatment order. For NaOCl, the lowest ratio was achieved when plasma treatment was applied prior to NaOCl treatment. The highest reduction was achieved by H_2_O_2_.

To our knowledge there are no studies that have investigated the combined effects of agents and plasma. To understand our observed results, we limit the discussion to studies that combined the agents with UV, ozone, and radicals. According to different mechanisms, agents used in this study can be classified into four groups: antiseptics (CHX, PHMB, and OCT), NaOCl, EDTA, and H_2_O_2_. With all agents, plasma interacts by the three main plasma constituents: UV radiation, ozone, and radicals. For each agent different mechanisms may be responsible for the increased effectivity of the combination of agent and plasma.

Firstly, no information has been published about interactions between radicals or radiation and antiseptics (CHX, PHMB, and OCT). After enquiry, the manufacturers confirmed UV stability of the products. Our spectral measurement of the antiseptics exposed before and after plasma treatment affirmed their information: antiseptics were not destroyed by the plasma treatment regime in our experiments. The antimicrobial efficacy of antiseptics was not decreased by additional plasma treatment as measured by CFU. For saliva biofilms the combination exceeded the pure plasma effect, which was corroborated by the live-dead staining experiment. Both treatment orders increased the antimicrobial effect, while prior plasma treatment tended to somewhat stronger antimicrobial effects. For OCT there was a significant difference between the two treatment orders: the biofilm could be destroyed by plasma so that the antiseptic could be more effective. Therefore, we might conclude that plasma does not destroy the antiseptics but rather has an additive effect in antiseptic treatment. For future medical therapies the combination of antiseptics and plasma treatment might be beneficial. 

It was shown that the combination of UV and NaOCl increased the inactivation of some phages, but without any synergistic effects concerning *E.coli* and enterococci in waste water [17]. The synergistic effect between hypochlorite and radicals increases protein damage [18], which can be relevant for the increase in antimicrobial effects. Because of the high antimicrobial efficacy of NaOCl in single treatment, combinations with Ar plasma lead to comparable log_10_CFUs for saliva biofilm (*P* > 0.05). Nevertheless, the combination with Ar plasma resulted in the highest CFU reduction factor, which was confirmed with Live-dead staining. The antimicrobial effect was not increased for NaOCl plus Ar plasma but for Ar plasma plus NaOCl. Regarding *S. mutans* biofilms and plaque our results are consistent.

Thirdly, the antibiofilm effect of EDTA is based on the dispersal of the biofilm structure [19]. Our working hypothesis was that after disruption of the biofilm matrix antimicrobial agents like plasma can kill the bacteria directly without being inhibited by the protective matrix. This explains why antibiotics can act more effectively with EDTA [20]. UV in combination with ozone or H_2_O_2_ is necessary for EDTA degradation, whereas pure ozone did not alter the action EDTA [21]. In plasma processes UV and ozone are developed. The resulting OH radicals degrade EDTA without creating toxic degradation products [22]. It is possible that EDTA was destroyed by plasma and the main effect of this combination is the dispersion of the biofilms by EDTA. EDTA showed per se an antimicrobial effect which could be increased using plasma. For *S. mutans* und saliva, Ar plasma plus EDTA was significantly more effective than Ar plasma or EDTA alone. According to our hypothesis, this synergistic effect is a consequence of the biofilm dispersion and the resulting better efficacy of plasma. To confirm this hypothesis, we changed the treatment order: first plasma, then EDTA. This order resulted in the same efficacy as that using CFU analysis. Using Live-dead staining we found only minimal differences between EDTA and plasma plus EDTA but a higher reduction of the live-dead ratio applying EDTA plus plasma (*P* < 0.05). This confirmed our hypothesis in agreement with other research groups who have combined EDTA with antibiotics [20]. 

Fourthly, the synergistic UV-H_2_O_2_ effect is already known and is used to disinfect food packaging materials [23] and to clean waste water [24]. The mechanism is based on the photolysis of H_2_O_2_. The reaction is induced by the absorption of photons by hydrogen peroxide, which leads to the production of OH radicals [25]. According to our hypothesis the additionally formed radicals support antimicrobial effects of H_2_O_2_ and plasma. To test this hypothesis, we switched the treatment order of H_2_O_2_ and plasma too. We found the same synergistic reduction effect after switching the order using determination of CFU and Live-dead staining, whereas a prior H_2_O_2_ treatment tended to increase antimicrobial effects. 

The antimicrobial effect of plasma may be attributed to a complex interaction of its components, for example, UV, radicals, ozone, and so forth. However, so far only single components such as UV have been combined with agents [18, 23, 26]. The current results deliver further information to understand antimicrobial effects of plasma in combination with agents as compared with effects of combinations between UV and agents because here a mixture of UV, radicals, and so forth was effective. 

There are some studies which determined the cytotoxic potential of single treatments with plasma and agents [27–29]. The toxic potential of the combination of plasma and agents needs further clarification. More investigations are necessary to identify the underlying mechanisms.

We performed *in vitro* studies. However, *in vivo* studies are necessary to ensure that (i) antimicrobial effects are not restricted to *in vitro* settings and (ii) application of combined treatments with Ar plasma and agents in clinical settings is reasonable. 

This is the first study evaluating the combined antimicrobial effect of plasma with different agents using three different biofilm models. The combination of plasma and agents increased the antimicrobial efficacy of all tested compounds. It supports the additional use of plasma treatment of dental implants in addition to the often used chemicals and antiseptic solutions. Thus, the combined treatment with plasma and agents seems very beneficial, since the efficacy will be increased, while no additional chemicals will be needed. Furthermore, our results indicate that even a reduction of commonly used agents could be possible when applied in combination with Ar plasma, which could reduce the costs for treatments as well as the possible risk for patients due to the antimicrobial agents. 

## Figures and Tables

**Table 1:** Logarithm of CFU/mL of *S. mutans*, saliva, and subgingival biofilms after treatment with different procedures (control compared with Ar gas or Ar plasma) and different agents (first agent then plasma).

Agent	*S. mutans *			Saliva			Plaque		
	Control	Ar gas	Ar plasma	Control	Ar gas	Ar plasma	Control	Ar gas	Ar plasma
NaCl control	6.91 ± 0.53 N = 16	6.56 ± 0.70 N = 8 RF = 0.35	4.80 ± 0.45^∗∗,#^ *N* = 15 RF = 2.11	7.39 ± 0.30 *N* = 32	6.98 ± 0.56 *N* = 16 RF = 0.41	5.91 ± 0.49^∗∗,#^ *N* = 38 RF = 1.48	7.33 ± 0.29 *N* = 21	6.00 ± 0.49** *N* = 14 RF = 1.33	5.17 ± 0.20^∗∗,#^ *N* = 14 RF = 2.16

CHX	5.13 ± 0.15* *N* = 8 RF = 1.78	4.95 ± 0.09* *N* = 8 RF = 1.96	4.96 ± 0.14 *N* = 8 RF = 1.95	7.11 ± 0.36 *N* = 24 RF = 0.28	6.01 ± 0.70^∗,∗∗^ *N* = 16 RF = 1.38	5.69 ± 0.65** *N* = 23 RF = 1.70	6.59 ± 0.42* *N* = 7 RF = 0.74	5.49 ± 0.12** *N* = 7 RF = 1.84	5.34 ± 0.05** *N* = 7 RF = 1.99

OCT	5.07 ± 0.16* *N* = 8 RF = 1.84	5.01 ± 0.36* *N* = 8 RF = 1.90	5.06 ± 0.12 *N* = 8 RF = 1.85	6.60 ± 0.58* *N* = 22 RF = 0.79	5.61 ± 0.57^∗,∗∗^ *N* = 13 RF = 1.78	4.60 ± 0.64^∗,∗∗,#^ *N* = 30 RF = 2.79	5.80 ± 0.33* *N* = 7 RF = 1.53	4.68 ± 1.53^∗,∗∗^ *N* = 7 RF = 2.65	5.34 ± 0.05 *N* = 7 RF = 1.99

PHMB	4.39 ± 0.22* **N** = 7 RF = 2.52	3.52 ± 0.66* *N* = 6 RF = 3.39	3.55 ± 0.24^∗,∗∗^ *N* = 7 RF = 3.36	6.52 ± 0.54* *N* = 16 RF = 0.87	6.26 ± 0.95 *N* = 8 RF = 1.13	5.83 ± 0.48** *N* = 23 RF = 1.56	6.37 ± 0.67* *N* = 7 RF = 0.96	5.47 ± 0.26 *N* = 7 RF = 1.86	5.26 ± 0.11 *N* = 7 RF = 2.07

NaOCl	0 ± 0* **N** = 7 **RF** = **6.91**	0 ± 0* **N** = 7 **RF** = **6.91**	0 ± 0* **N** = 8 **RF** = **6.91**	5.00 ± 0.11* *N* = 6 RF = 2.39	4.51 ± 0.60* *N* = 7 RF = 2.88	4.33 ± 0.31* **N** = 8 **RF** = **3.06**	5.57 ± 0.06* *N* = 7 RF = 1.76	5.59 ± 0.06 *N* = 7 RF = 1.74	5.69 ± 0.03 *N* = 7 RF = 1.64

H_2_O_2_	0 ± 0* **N** = 8 **RF** = **6.91**	0 ± 0* **N** = 7 **RF** = **6.91**	0 ± 0* **N** = 8 **RF** = **6.91**	5.90 ± 0.29* *N* = 8 RF = 1.49	5.56 ± 0.54* *N* = 7 RF = 1.83	4.98 ± 1.51 *N* = 8 RF = 2.41	5.45 ± 0.06* *N* = 7 RF = 1.88	5.49 ± 0.08 *N* = 7 RF = 1.84	4.41 ± 0.86^#^ **N** = 7 **RF** = **2.92**

EDTA	5.79 ± 0.22* *N* = 9 RF = 1.12	5.64 ± 0.32 *N* = 9 RF = 1.27	4.13 ± 0.32^∗,∗∗,#^ *N* = 7 RF = 2.78	6.76 ± 0.11* *N* = 8 RF = 0.63	6.67 ± 0.20 *N* = 8 RF = 0.72	4.58 ± 0.62^∗,∗∗,#^ *N* = 8 RF = 2.81	5.60 ± 0.30* *N* = 7 RF = 1.73	5.31 ± 0.10 *N* = 7 RF = 2.02	5.25 ± 0.18 *N* = 7 RF = 2.08

Data are presented as mean lg (CFU/mL + 1) ± standard deviation. Ar: argon; CHX: 0.1% chlorhexidine; OCT: 0.1% octenidine; PHMB: 0.1% polyhexanide; NaOCl: 0.6% sodium hypochlorite; H_2_O_2_: 1.5% hydrogen peroxide; EDTA: 20% ethylenediaminetetraacetic acid.

Entries with the lowest CFUs/mL were boldface font.

*P < 0.05 versus NaCl control (within columns); **P < 0.05 versus control procedure (within rows); ^#^ *P* < 0.05 Ar plasma versus Ar gas (within rows), two-sided Mann-Whitney *U* tests, and reduction in respective CFUs/mL. *P* values were Bonferroni corrected.

**Table 2:** Linear regression models evaluating effects of different agents and plasma procedures on lg (CFU/mL + 1) for *S. mutans*, saliva, and subgingival biofilms (first agent then plasma).

	*S. mutans* (*N* = 178, *R* ^2^ = 98.4%)		Saliva (*N* = 329, *R* ^2^ = 72.6%)		Subgingival (*N* = 175, *R* ^2^ = 76.3%)	
	*B* (95% CI)	*P *	*B* (95% CI)	*P *	*B* (95% CI)	*P *
Agent (ref. NaCl control)						
CHX	−1.78 (−2.06; −1.50)	<0.001	−0.29 (−0.59; 0.02)	<0.001	−0.74 (−1.12; −0.36)	<0.001
OCT	−1.84 (−2.12; −1.56)	<0.001	−0.79 (−1.10; −0.49)	<0.001	−1.54 (−1.91; −1.16)	<0.001
PHMB	−2.51 (−2.81; −2.22)	<0.001	−0.87 (−1.22; −0.53)	<0.001	−0.96 (−1.34; −0.58)	<0.001
NaOCl	−6.91 (−7.20; −6.61)	<0.001	−2.39 (−2.89; −1.90)	<0.001	−1.76 (−2.14; −1.38)	<0.001
H_2_O_2_	−6.91 (−7.19; −6.63)	<0.001	−1.50 (−1.94; −1.06)	<0.001	−1.89 (−2.27; −1.51)	<0.001
EDTA	−1.11 (−1.38; −0.84)	<0.001	−0.64 (−1.08; −0.19)	0.005	−1.74 (−2.12; −1.36)	<0.001

Procedure (ref. control)						
Ar gas	−0.35 (−0.63; −0.07)	0.02	−0.42 (−0.76; −0.07)	0.02	−1.34 (−1.64; −1.04)	<0.001
Ar plasma	−2.10 (−2.34; −1.87)	<0.001	−1.49 (−1.76; −1.22)	<0.001	−2.16 (−2.46; −1.86)	<0.001

Interaction agent × procedure						
CHX × Ar gas	0.18 (−0.25; 0.60)	0.42	−0.68 (−1.17; −0.19)	0.008	0.24 (−0.31; 0.79)	0.40
OCT × Ar gas	0.29 (−0.14; 0.71)	0.19	−0.58 (−1.10; −0.61)	0.03	0.22 (−0.33; 0.78)	0.43
PHMB × Ar gas	−0.53 (−0.99; −0.07)	0.02	0.15 (−0.44; 0.75)	0.61	0.44 (−0.12; 0.99)	0.12
NaOCl × Ar gas	0.35 (−0.10; 0.79)	0.12	−0.07 (−0.78; 0.64)	0.84	1.35 (0.80; 1.91)	<0.001
H_2_O_2_ × Ar gas	0.35 (−0.09; 0.78)	0.12	0.08 (−0.59; 0.75)	0.81	1.38 (0.83; 1.94)	<0.001
EDTA × Ar gas	0.19 (−0.22; 0.61)	0.36	0.32 (−0.33; 0.98)	0.33	1.05 (0.50; 1.61)	<0.001
CHX × Ar plasma	1.93 (1.53; 2.33)	<0.001	0.08 (−0.34; 0.50)	0.72	0.91 (0.35; 1.46)	0.001
OCT × Ar plasma	2.09 (1.70; 2.49)	<0.001	−0.51 (−0.92; −0.10)	0.02	1.70 (1.15; 2.25)	<0.001
PHMB × Ar plasma	1.26 (0.85; 1.68)	<0.001	0.80 (0.35; 1.25)	0.001	1.04 (0.49; 1.60)	<0.001
NaOCl × Ar plasma	2.10 (1.70; 2.51)	<0.001	0.81 (0.16; 1.47)	0.02	2.27 (1.72; 2.82)	<0.001
H_2_O_2_ × Ar plasma	2.10 (1.70; 2.50)	<0.001	0.57 (−0.05; 1.19)	0.07	1.12 (0.57; 1.67)	<0.001
EDTA × Ar plasma	0.44 (0.04; 0.84)	0.03	−0.69 (−1.31; −0.08)	0.03	1.82 (1.26; 2.37)	<0.001

Ar: argon; CHX: 0.1% chlorhexidine; OCT: 0.1% octenidine; PHMB: 0.1% polyhexanide; NaOCl: 0.6% sodium hypochlorite; H_2_O_2_: 1.5% hydrogen peroxide; EDTA: 20% ethylenediaminetetraacetic acid.

**Table 3:** Logarithm of saliva biofilm CFU/mL after treatment with different procedures (control compared with Ar gas or Ar plasma) and different agents (NaCl, H_2_O_2_, and EDTA) in changed order (first plasma then agent treatment).

Agent	Procedure		
	Control	Ar gas	Ar plasma
NaCl control	7.17 ± 0.48 *N* = 21	6.59 ± 0.48** *N* = 15	5.82 ± 0.52^∗∗,#^ *N* = 8
H_2_O_2_	6.17 ± 0.80* *N* = 14	5.62 ± 0.86* *N* = 15	4.59 ± 0.77^∗,∗∗,#^ *N* = 11
EDTA	5.92 ± 0.37* *N* = 8	6.25 ± 0.43** *N* = 14	4.51 ± 0.82^∗,∗∗,#^ *N* = 16

Data are presented as mean lg (CFU/mL + 1) ± SD.

Ar: argon; CHX: 0.1% chlorhexidine; OCT: 0.1% octenidine; PHMB: 0.1% polyhexanide; NaOCl: 0.6% sodium hypochlorite; H_2_O_2_: 1.5% hydrogen peroxide; EDTA: 20% ethylenediaminetetraacetic acid.

*P < 0.05 versus NaCl control (within columns); **P < 0.05 versus control procedure (within rows); ^#^ *P* < 0.05 comparing Ar plasma versus Ar gas (within rows), two-sided Mann-Whitney *U* test, and reduction in saliva CFU/mL. *P* values were Bonferroni corrected.

## References

[1] Morfill GE, Kong MG, Zimmermann JL (2009). Focus on plasma medicine. *New Journal of Physics*.

[2] Stoffels E, Flikweert AJ, Stoffels WW, Kroesen GMW (2002). Plasma needle: a non-destructive atmospheric plasma source for fine surface treatment of (bio)materials. *Plasma Sources Science and Technology*.

[3] Kramer A, Assadian O, Below H (2010). Perspektiven der Plasmamedizin. *Vakuum in Forschung Und Praxis*.

[4] Koban I, Duske K, Jablonowski L (2011). Atmospheric plasma enhances wettability and osteoblast spreading on dentin in vitro: proof-of-principle. *Plasma Processes and Polymers*.

[5] Koban I, Holtfreter B, Hübner N-O (2011). Antimicrobial efficacy of non-thermal plasma in comparison to chlorhexidine against dental biofilms on titanium discs in vitro—proof of principle experiment. *Journal of Clinical Periodontology*.

[6] Koban I, Matthes R, Hübner N-O (2010). Treatment of Candida albicans biofilms with low-temperature plasma induced by dielectric barrier discharge and atmospheric pressure plasma jet. *New Journal of Physics*.

[7] Fridman G, Friedman G, Gutsol A, Shekhter AB, Vasilets VN, Fridman A (2008). Applied plasma medicine. *Plasma Processes and Polymers*.

[8] Weltmann K-D, Kindel E, Brandenburg R (2009). Atmospheric pressure plasma jet for medical therapy: plasma parameters and risk estimation. *Contributions to Plasma Physics*.

[9] Halfmann H, Bibinov N, Wunderlich J, Awakowicz P (2007). A double inductively coupled plasma for sterilization of medical devices. *Journal of Physics D*.

[10] Halfmann H, Denis B, Bibinov N, Wunderlich J, Awakowicz P (2007). Identification of the most efficient VUV/UV radiation for plasma based inactivation of Bacillus atrophaeus spores. *Journal of Physics D*.

[11] Merritt J, Qi F, Goodman SD, Anderson MH, Shi W (2003). Mutation of luxS affects biofilm formation in Streptococcus mutans. *Infection and Immunity*.

[12] Fujioka-Hirai Y, Akagawa Y, Minagi S (1987). Adherence of Streptococcus mutans to implant materials. *Journal of Biomedical Materials Research*.

[13] Netuschil L, Brecx M, Vohrer KG, Riethe P (1996). Vital fluorescence to assess in vitro and in vivo the antibacterial effects of amalgams. *Acta stomatologica Belgica*.

[14] Müller G, Winkler Y, Kramer A (2003). Antibacterial activity and endotoxin-binding capacity of Actisorb Silver 220. *Journal of Hospital Infection*.

[15] Filoche SK, Sissons CH, Sladek REJ, Stoffels E, Güceri S, Fridman, A (2008). Cold plasma treatment of in vitro dental plaque. *Plasma Assisted Decontamination of Biological and Chemical Agents*.

[16] Bogosian G, Bourneuf EV (2001). A matter of bacterial life and death. *EMBO Reports*.

[17] Montemayor M, Costan A, Lucena F (2008). The combined performance of UV light and chlorine during reclaimed water disinfection. *Water Science and Technology*.

[18] Hawkins CL, Rees MD, Davies MJ (2002). Superoxide radicals can act synergistically with hypochlorite to induce damage to proteins. *FEBS Letters*.

[19] Banin E, Brady KM, Greenberg EP (2006). Chelator-induced dispersal and killing of Pseudomonas aeruginosa cells in a biofilm. *Applied and Environmental Microbiology*.

[20] Raad I, Chatzinikolaou I, Chaiban G (2003). In vitro and ex vivo activities of minocycline and EDTA against microorganisms embedded in biofilm on catheter surfaces. *Antimicrobial Agents and Chemotherapy*.

[21] Rodríguez JB, Mutis A, Yeber MC, Freer J, Baeza J, Mansilla HD (1999). Chemical degradation of EDTA and DTPA in a totally chlorine free (TCF) effluent. *Water Science and Technology*.

[22] Sorensen M, Zurell S, Frimmel FH (1998). Degradation pathway of the photochemical oxidation of ethylenediaminetetraacetate (EDTA) in the UV/H2O2-process. *Acta Hydrochimica et Hydrobiologica*.

[23] Reidmiller JS, Baldeck JD, Rutherford GC, Marquis RE (2003). Characterization of UV-peroxide killing of bacterial spores. *Journal of Food Protection*.

[24] Chang PBL, Young TM (2000). Kinetics of methyl tert-butyl ether degradation and by-product formation during UV/hydrogen peroxide water treatment. *Water Research*.

[25] Johansson O, Bood J, Aldén M, Lindblad U (2009). Hydroxyl radical consumption following photolysis of vapor-phase hydrogen peroxide at 266 nm: Implications for photofragmentation laser-induced fluorescence measurements of hydrogen peroxide. *Applied Physics B*.

[26] Zeng Q-F, Fu J, Shi Y-T, Xia D-S, Zhu H-L (2009). Adsorbable organic halogens generation and reduction during degradation of phenol by UV radiation/sodium hypochlorite. *Water Environment Research*.

[27] Bender C, Matthes R, Kindel E (2010). The irritation potential of nonthermal atmospheric pressure plasma in the HET-CAM. *Plasma Processes and Polymers*.

[28] Müller G, Kramer A (2008). Biocompatibility index of antiseptic agents by parallel assessment of antimicrobial activity and cellular cytotoxicity. *Journal of Antimicrobial Chemotherapy*.

[29] Müller G, Kramer A, Siebert J (2009). Effectiveness of octenidine and chlorhexidine in the artificially contaminated 3-D-culture of human keratinocytes. *GMS Krankenhaushyg Interdiszip*.

